# Plasma fibrinogen and mortality in patients undergoing peritoneal dialysis: a prospective cohort study

**DOI:** 10.1186/s12882-020-01984-6

**Published:** 2020-08-17

**Authors:** Jing Yu, Tong Lin, Naya Huang, Xi Xia, Jianbo Li, Yagui Qiu, Xiao Yang, Haiping Mao, Fengxian Huang

**Affiliations:** 1 Department of Nephrology, The First Affiliated Hospital, Sun Yat-sen University, Guangzhou, Guangdong Province China; 2 Key Laboratory of Nephrology, National Health Commission and Guangdong Province, The First Affiliated Hospital, Sun Yat-sen University, Guangzhou, Guangdong Province China

**Keywords:** Plasma fibrinogen, Peritoneal dialysis, Cardiovascular mortality, All-cause mortality

## Abstract

**Background:**

Plasma fibrinogen is significantly associated with cardiovascular (CV) events and mortality in the general population. However, the association between plasma fibrinogen and mortality in patients undergoing peritoneal dialysis (PD) is unclear.

**Methods:**

This was a prospective cohort study. A total of 1603 incident PD patients from a single center in South China were followed for a median of 46.7 months. A Cox regression analysis was used to evaluate the independent association of plasma fibrinogen with CV and all-cause mortality. Models were adjusted for age, sex, smoking, a history of CV events, diabetes, body mass index, systolic blood pressure, hemoglobin, blood platelet count, serum potassium, serum albumin, low-density lipoprotein cholesterol, high-density lipoprotein cholesterol, hypersensitive C-reactive protein, estimated glomerular filtration rate, antiplatelet agents and lipid-lowering drugs.

**Results:**

The mean age was 47.4 ± 15.3 years, 955 (59.6%) patients were male, 319 (19.9%) had a history of CV events, and 410 (25.6%) had diabetes. The average plasma fibrinogen level was 4.12 ± 1.38 g/L. Of the 474 (29.6%) patients who died during follow-up, 235 (49.6%) died due to CV events. In multivariable models, the adjusted hazard ratios (HRs) for quartile 1, quartile 3, and quartile 4 versus quartile 2 were 1.18 (95% confidence interval [CI], 0.72–1.95, *P* = 0.51), 1.47 (95% CI, 0.93–2.33, *P* = 0.10), and 1.78 (95% CI, 1.15–2.77, *P* = 0.01) for CV mortality and 1.20 (95% CI, 0.86–1.68, *P* = 0.28), 1.29 (95% CI, 0.93–1.78, *P* = 0.13), and 1.53 (95% CI, 1.12–2.09, *P* = 0.007) for all-cause mortality, respectively. A nonlinear relationship between plasma fibrinogen and CV and all-cause mortality was observed.

**Conclusions:**

An elevated plasma fibrinogen level was significantly associated with an increased risk of CV and all-cause mortality in patients undergoing PD.

## Background

Patients with end-stage renal disease (ESRD) exhibit a high risk for cardiovascular (CV) event morbidity and mortality [1]. The high prevalence of diabetes, hypertension, dyslipidemia, and other traditional CV risk factors only partially explains the high CV risk of these individuals [2]. Some nontraditional CV risk factors associated with the inflammation-coagulation axis, such as hypersensitive C-reactive protein (hs-CRP), hyperhomocysteinemia, and increased prevalence of abnormal altered coagulation with declining renal function, have been reported to be new strong CV risk factors [3–5]. Plasma fibrinogen plays an important role in the coagulation cascade; levels that are too high or too low may cause an increased risk of thrombosis and bleeding, respectively. Studies have shown that the fibrinogen level is influenced by some traditional CV risk factors, such as age, smoking, diabetes, and hypertension, as well as emerging risk factors, such as inflammation [6, 7]. Therefore, fibrinogen may not only represent a risk factor [8] but also provide a common pathway for the interaction of various risk factors promoting CV events.

Fibrinogen is an independent risk factor of CV events and mortality in the general population [9, 10]. However, it has been reported that uremia has a negative effect on hemostasis, which is called uremic coagulopathy. Uremic coagulopathy causes increased production and/or decreased clearance of procoagulant proteins, leading to increased fibrinogen levels [11–13]. ESRD patients are not a homogeneous cohort, as they include patients undergoing hemodialysis (HD) and peritoneal dialysis (PD). Thus, the coagulation profile of these individuals is not completely the same, which makes the relationship between fibrinogen and mortality complicated and controversial. Elevated plasma fibrinogen independently predicts CV events in individuals with stage 3–4 chronic kidney disease (CKD) [14]. However, the study conducted by Shlipak et al. [15] found that fibrinogen could not predict adverse outcomes. Zoccali et al. [16] found a positive association between high plasma fibrinogen and mortality in HD patients, but this has been contradicted by other studies conducted on HD patients [17, 18]. Patients with PD can have metabolic abnormalities such as insulin resistance, dyslipidemia, and metabolic syndrome due to long-term exposure to glucose-based dialysate, which links endothelial dysfunction, inflammation, and a tendency toward procoagulation together [19]. Therefore, patients on PD have higher fibrinogen levels and a more prothrombotic profile than patients on HD [20, 21]. We hypothesized that elevated plasma fibrinogen levels are significantly associated with mortality in PD patients. Until now, research on the association between plasma fibrinogen and mortality in PD patients has been limited, and the sample sizes of these studies have been relatively small [22, 23]. Thus, the aim of this study was to examine the association of plasma fibrinogen with CV and all-cause mortality in a large cohort of PD patients.

## Methods

### Participants

From January 1, 2006, to December 31, 2013, we enrolled 1955 patients who initiated PD therapy at a single PD center of the First Affiliated Hospital of Sun Yat-sen University in South China. Eligible patients included those older than 18 years who had undergone catheterization for PD at our center, received PD for more than 3 months, and signed informed consent. We excluded patients who had undergone long-term HD (more than 3 months), those who had received a kidney transplant, those with malignant tumors, those who lacked the baseline fibrinogen data, and those who had an outlier plasma fibrinogen concentration <  0.5th or > 99.5th percentile of the observed values. In the end, we included 1603 patients and followed up with them until August 31, 2018.

### Data collection and study protocol

This was a prospective cohort study conducted at our PD center. Baseline demographic data, including age, sex, smoking, a history of CV events, diabetes, and hypertension, were collected at the initiation of PD therapy. A history of CV events was defined as a patient who had one or more of the following CV events: angina, myocardial infarction, heart failure, angioplasty, coronary artery bypass or stroke. Diabetic patients were those who met the clinical diagnostic criteria for diabetes mellitus and/or those who currently or previously used insulin or oral hypoglycemic agents. Hypertensive patients were those who had at least two separate blood pressure measurements above 140/90 mmHg and/or those who used antihypertensive drugs currently or previously.

Clinical and biochemical data, including body mass index (BMI), blood pressure, medication use, plasma fibrinogen, hemoglobin, blood platelet count, serum potassium, serum albumin, serum creatinine, total cholesterol (TC), triglycerides (TG), low-density lipoprotein cholesterol (LDL-C), high-density lipoprotein cholesterol (HDL-C), and hs-CRP levels, were collected 3 months after PD therapy initiation. The blood samples were all measured at the same Inspection Center of the First Affiliated Hospital of Sun Yat-sen University. Plasma fibrinogen was measured by a commercial assay reagent (Dade Thrombin Reagent, Siemens, Germany). The normal range of plasma fibrinogen measured in our hospital was 2–4 g/L. The estimated glomerular filtration rate (eGFR) was used to evaluate baseline residual renal function, which was calculated by the Chronic Kidney Disease Epidemiology Collaboration (CKD-EPI) equation. In the process of PD, the dextrose concentration of peritoneal dialysate was usually 1.5% or 2.5%.

Medication usage data were derived from prescriptions. Antiplatelet agents included aspirin, clopidogrel, and dipyridamole. Lipid-lowering drugs included statins and fibrates. Patients were requested to return to our center quarterly for a comprehensive medical assessment and were interviewed by telephone or face to face monthly by trained nurses and physicians to assess their general condition and adjust the use of medications.

The primary outcome of this study was CV mortality; the secondary outcome was all-cause mortality. CV mortality referred to death from acute myocardial infarction, atherosclerotic heart disease, congestive heart failure, cardiac arrhythmia, sudden death, cardiomyopathy, ischemic or hemorrhagic stroke, and peripheral vascular disease. In the case of in-hospital death, death certificates were used to identify the exact cause of death. In the case of out-of-hospital death, family members were interviewed by telephone to ascertain the circumstances of death. Subsequently, the cause of death was determined by study physicians based on a series of comprehensive considerations of signs and symptoms before and after death, the history and recent health status, and descriptions provided by the patient’s family members. All patients were followed up until death, kidney transplantation, transfer to HD therapy, transfer to other centers, loss to follow-up, or the end of follow-up on August 31, 2018. This study was conducted in compliance with the principles of the Declaration of Helsinki and was approved by the Clinical Research Ethics Committee of the First Affiliated Hospital of Sun Yat-sen University. All patients signed informed consent before they entered the study.

### Statistical analysis

The study population was divided into quartiles according to plasma fibrinogen levels: quartile 1, < 3.19; quartile 2, 3.19–3.80; quartile 3, 3.80–4.72; and quartile 4, > 4.72. Quantitative data were tested for normality by the Kolmogorov-Smirnov test, and data were considered normally distributed if the *P* value was > 0.05. Data are described as means ± standard deviations for normally distributed continuous variables, as medians (interquartile ranges, 1/4–3/4) for non-normally distributed continuous variables, and as frequencies (percentages) for categorical data. Comparisons of clinical variables according to plasma fibrinogen quartiles were performed using one-way analysis of variance (ANOVA), the Kruskal-Wallis test, or the chi-squared test according to appropriate corresponding data types. A multiple linear regression was used to explore the independent associated factors of plasma fibrinogen levels, which were adjusted for significant covariates in a simple linear regression using a stepwise conditional method.

Survival was analyzed by the Kaplan-Meier method, and differences in survival distributions among plasma fibrinogen quartiles were evaluated by a Mantel (log-rank) test. A multivariable Cox proportional hazards method was used to evaluate the independent association of plasma fibrinogen levels with CV and all-cause mortality.

First, plasma fibrinogen was divided into quartiles. Given that increased or decreased fibrinogen levels may be associated with CV and all-cause mortality, we chose the second quartile (Q2) as the reference. In the multivariable model, model 1 adjusted for basic covariates (age and sex). Model 2 included model 1 covariates plus comorbid conditions (smoking, a history of CV events, diabetes, BMI, and systolic blood pressure). Model 3 included model 2 covariates plus biochemical data (hemoglobin, blood platelet count, serum potassium, serum albumin, LDL-C, HDL-C, hs-CRP, and eGFR). Model 4 included model 3 covariates and treatments (antiplatelet agents and lipid-lowering drugs). We used the hazard ratio (HR) and 95% confidence interval (CI) to describe the results.

Second, we explored the continuous, potentially nonlinear, relationship between plasma fibrinogen and mortality by using fully adjusted restricted cubic spline models. *P <* 0.05 was considered statistically significant. Statistical analyses were performed using SPSS software (SPSS, version 13.0, IBM Corp., Chicago, IL, United States) and STATA software (STATA, version 14.0, Stata Corp LP, College Station, TX, United States).

## Results

### Participants

**Fig. 1:**
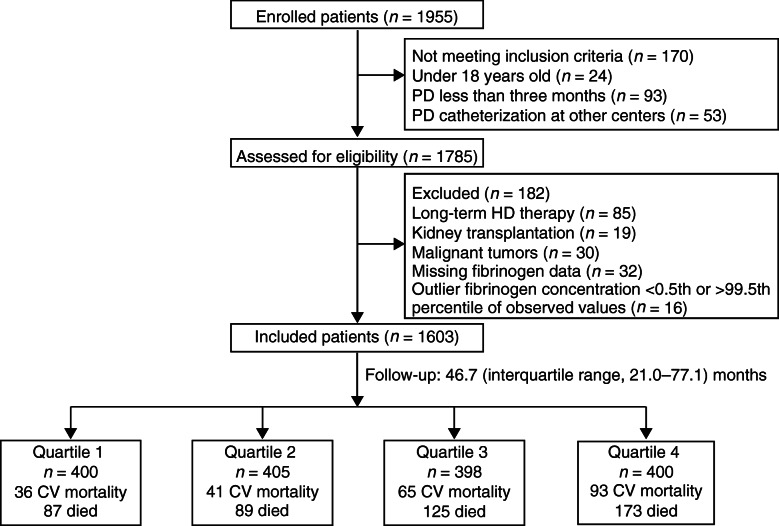
Enrollment flow chart for analysis. *Abbreviations: PD* peritoneal dialysis, *HD* hemodialysis, *CV* cardiovascular

A total of 1955 patients undergoing PD were enrolled at a single PD center. In the end, 1603 incident PD patients were finally included in this study and followed for a median of 46.7 (maximum, 152.5) months (Fig. 1). The baseline characteristics of the study patients are shown in Table 1. Their mean age was 47.4 ± 15.3 years, 955 (59.6%) were male, 319 (19.9%) had a history of CV events, and 410 (25.6%) had diabetes. The average plasma fibrinogen level was 4.12 ± 1.38 g/L. Older age, a higher proportion of smoking, a history of CV events, diabetes, and the use of antiplatelet agents and lipid-lowering drugs were prominent in higher quartiles. Moreover, with regard to biochemical data, patients with higher plasma fibrinogen levels had higher blood platelet counts, TC, TG, LDL-C, and hs-CRP levels but lower serum albumin and HDL-C levels.

**Table 1:** Baseline characteristics of the study cohort according to plasma fibrinogen quartiles

Variables	Total (*n* = 1603)	Plasma fibrinogen quartiles				*P*
		Q1 (< 3.19) (*n* = 400)	Q2 (3.19–3.80) (*n* = 405)	Q3 (3.80–4.72) (*n* = 398)	Q4 (> 4.72) (*n* = 400)	
Plasma fibrinogen (g/L)	4.12 ± 1.38	2.72 ± 0.35	3.48 ± 0.18	4.26 ± 0.27	6.04 ± 1.16	< 0.001
Age (y)	47.4 ± 15.3	43.9 ± 14.9	45.9 ± 15.6	47.8 ± 14.5	52.1 ± 14.8	< 0.001
Male, n (%)	955 (59.6)	220 (55.0)	234 (57.8)	245 (61.6)	256 (64.0)	0.05
Smokers, n (%)	331 (20.6)	65 (16.3)	76 (18.8)	81 (20.4)	109 (27.3)	0.001
History of CV events, n (%)	319 (19.9)	47 (11.8)	61 (15.1)	89 (22.4)	122 (30.5)	< 0.001
Diabetes, n (%)	410 (25.6)	49 (12.3)	79 (19.5)	114 (28.6)	168 (42.0)	< 0.001
Hypertension, n (%)	1416 (88.3)	353 (88.3)	358 (88.4)	356 (89.4)	349 (87.3)	0.82
BMI (kg/m^2^)	22.04 ± 3.29	21.15 ± 2.78	21.66 ± 3.15	22.31 ± 3.19	23.07 ± 3.68	< 0.001
SBP (mmHg)	154.3 ± 24.8	150.4 ± 24.4	154.8 ± 23.7	157.3 ± 27.2	154.8 ± 23.5	0.002
DBP (mmHg)	88.4 ± 15.6	88.4 ± 16.3	88.6 ± 14.8	89.6 ± 16.1	86.7 ± 15.3	0.07
Hemoglobin (g/L)	79.1 ± 18.6	78.9 ± 19.1	77.9 ± 18.9	80.0 ± 18.6	79.8 ± 18.0	0.36
Blood platelet count (10 × 10^9^/L)	19.3 ± 8.0	16.5 ± 6.6	17.7 ± 7.2	19.8 ± 7.5	23.4 ± 8.9	< 0.001
Serum potassium (mmol/L)	4.65 ± 0.84	4.69 ± 0.83	4.73 ± 0.80	4.68 ± 0.84	4.50 ± 0.86	< 0.001
Serum albumin (g/L)	34.8 ± 5.1	35.9 ± 5.1	35.1 ± 4.7	34.7 ± 5.0	33.3 ± 5.3	< 0.001
TC (mmol/L)	4.72 ± 1.43	4.44 ± 1.31	4.53 ± 1.13	4.91 ± 1.60	4.98 ± 1.57	< 0.001
TG (mmol/L)	1.30 (0.93–1.88)	1.15 (0.80–1.61)	1.26 (0.94–1.84)	1.32 (0.97–1.88)	1.49 (1.02–2.17)	< 0.001
LDL-C (mmol/L)	2.83 ± 1.09	2.59 ± 0.97	2.69 ± 0.85	3.00 ± 1.23	3.05 ± 1.19	< 0.001
HDL-C (mmol/L)	1.05 ± 0.33	1.11 ± 0.34	1.04 ± 0.32	1.05 ± 0.33	1.00 ± 0.31	< 0.001
hs-CRP (mg/L)	2.23 (0.74–8.49)	1.16 (0.44–4.08)	1.66 (0.49–6.29)	2.91 (1.01–9.50)	5.09 (1.47–11.79)	< 0.001
eGFR (mL/min/1.73 m^2^)	5.2 ± 2.4	5.3 ± 2.4	5.1 ± 2.2	5.2 ± 2.6	5.3 ± 2.5	0.62
Antiplatelet agents, n (%)	182 (11.4)	25 (6.3)	38 (9.4)	46 (11.6)	73 (18.3)	< 0.001
Lipid-lowering drugs, n (%)	182 (11.4)	23 (5.8)	40 (9.9)	49 (12.3)	70 (17.5)	< 0.001

*Note:* Continuous quantitative variables are described as means ± standard deviations or medians (interquartile ranges, 1/4–3/4). Categorical data are described as frequencies (percentages)

*Abbreviations: Q1–Q4* lowest to highest quartile, *CV* cardiovascular, *BMI* body mass index, *SBP* systolic blood pressure, *DBP* diastolic blood pressure, *TC* total cholesterol, *TG* triglycerides, *LDL-C* low-density lipoprotein cholesterol, *HDL-C* high-density lipoprotein cholesterol, *hs-CRP* hypersensitive C-reactive protein, *eGFR* estimated glomerular filtration rate

At the end of the follow-up, 351 (21.9%) patients continued PD treatment, 368 (23.0%) underwent kidney transplantation, 273 (17.0%) transferred to HD treatment, 75 (4.7%) transferred to other centers, and 62 (3.9%) were lost to follow-up. We recorded 474 (29.6%) deaths, of which 235 (49.7%) were due to CV events. Other causes of death were as follows: 99 (20.9%), infection; 13 (2.7%), malignant tumor; 13 (2.7%), gastrointestinal hemorrhage; 22 (4.6%), failure/dyscrasia; 11 (2.3%), giving up treatment; 51 (10.8%), unknown causes; and 30 (6.3%), other causes.

### Independent factors associated with plasma fibrinogen levels

Table 2 shows independent associated factors of plasma fibrinogen levels. These values were obtained by adjusting for significant covariates in a simple linear regression (Additional file 1: Table S1) and using a stepwise selection procedure. Plasma fibrinogen levels were independently positively linearly associated with age (β = 0.07, *P* = 0.01), diabetes (β = 0.08, *P* = 0.005), BMI (β = 0.07, *P* = 0.009), blood platelet count (β = 0.24, *P* <  0.001), LDL-C (β = 0.10, *P* <  0.001), and hs-CRP (β = 0.19, *P* <  0.001), while negatively linearly associated with serum potassium (β = − 0.08, *P* = 0.001), serum albumin (β = − 0.12, *P* <  0.001), and HDL-C (β = − 0.07, *P* = 0.004).

**Table 2:** Independent associated factors of plasma fibrinogen levels in a multiple linear regression model

Variables	Unstandardized coefficients		Standardized coefficients	t	*P*
	B	Standard error	β		
Age (years)	0.006	0.002	0.07	2.46	0.01
Diabetes (yes/no)	0.25	0.09	0.08	2.79	0.005
BMI (kg/m^2^)	0.03	0.01	0.07	2.62	0.009
Blood platelet count (per 10 × 10^9^/L greater)	0.04	0.004	0.24	9.31	< 0.001
Serum potassium (mmol/L)	−0.13	0.04	−0.08	−3.35	0.001
Serum albumin (g/L)	−0.03	0.007	−0.12	−4.66	< 0.001
LDL-C (mmol/L)	0.12	0.03	0.10	3.77	< 0.001
HDL-C (mmol/L)	−0.30	0.10	−0.07	−2.89	0.004
hs-CRP (per log-unit greater)	0.18	0.02	0.19	7.32	< 0.001

*Note:* Analysis was performed to explore the independent factors linearly associated with plasma fibrinogen levels in a multiple linear regression model. *F =* 47.31, *P* <  0.001, *R*^2^ = 0.243, adjusted *R*^2^ = 0.237. Covariates for adjustment are listed in Additional file 1: Table S1, stepwise conditional adjustment. Hypersensitive C-reactive protein was log-transformed

*Abbreviations: BMI* body mass index, *LDL-C* low-density lipoprotein cholesterol, *HDL-C* high-density lipoprotein cholesterol, *hs-CRP* hypersensitive C-reactive protein

### Plasma fibrinogen and CV and all-cause mortality

**Fig. 2:**
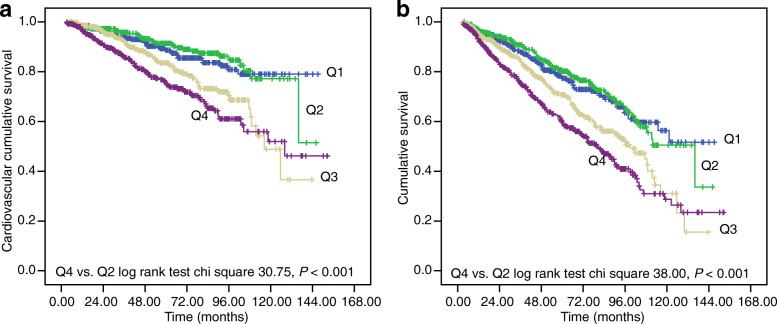
Cardiovascular and all-cause mortality-free survival curves according to plasma fibrinogen quartiles. **a** Cardiovascular mortality-free survival curves according to plasma fibrinogen quartiles. **b** All-cause mortality-free survival curves according to plasma fibrinogen quartiles. *Abbreviations: Q1 to Q4* lowest to highest quartile

Kaplan-Meier survival curves of plasma fibrinogen quartiles are shown in Fig. 2. CV and all-cause mortality (Fig. 2a and b, respectively) in patients in quartile 4 were significantly higher than those in quartile 2 (log-rank test *P <* 0.001, for both CV and all-cause mortality).

Table 3 lists the association between plasma fibrinogen and CV and all-cause mortality. In multivariable model 4, the adjusted HRs for quartile 1, quartile 3, and quartile 4 versus quartile 2 were 1.18 (95% CI, 0.72–1.95, *P* = 0.51), 1.47 (95% CI, 0.93–2.33, *P* = 0.10), and 1.78 (95% CI, 1.15–2.77, *P* = 0.01) for CV mortality and 1.20 (95% CI, 0.86–1.68, *P* = 0.28), 1.29 (95% CI, 0.93–1.78, *P* = 0.13), and 1.53 (95% CI, 1.12–2.09, *P* = 0.007) for all-cause mortality, respectively.

**Table 3:** Association of plasma fibrinogen quartiles with CV and all-cause mortality

Variables	Q1 (*n* = 400)		Q3 (*n* = 398)		Q4 (*n* = 400)	
	HR (95% CI)	*P*	HR (95% CI)	*P*	HR (95% CI)	*P*
CV mortality						
Unadjusted	0.86 (0.55–1.34)	0.49	1.74 (1.18–2.58)	0.005	2.43 (1.68–3.51)	< 0.001
Model 1^a^	0.92 (0.59–1.44)	0.71	1.58 (1.07–2.34)	0.02	1.93 (1.33–2.79)	< 0.001
Model 2^b^	1.04 (0.65–1.64)	0.89	1.42 (0.94–2.13)	0.09	1.74 (1.19–2.55)	0.004
Model 3^c^	1.18 (0.72–1.94)	0.52	1.46 (0.92–2.32)	0.11	1.76 (1.13–2.72)	0.01
Model 4^d^	1.18 (0.72–1.95)	0.51	1.47 (0.93–2.33)	0.10	1.78 (1.15–2.77)	0.01
All-cause mortality						
Unadjusted	0.95 (0.71–1.28)	0.74	1.54 (1.18–2.03)	0.002	2.09 (1.62–2.70)	< 0.001
Model 1^a^	1.00 (0.75–1.35)	0.99	1.41 (1.08–1.86)	0.01	1.67 (1.29–2.16)	< 0.001
Model 2^b^	1.08 (0.80–1.47)	0.60	1.26 (0.95–1.67)	0.11	1.45 (1.11–1.89)	0.006
Model 3^c^	1.19 (0.85–1.67)	0.30	1.28 (0.93–1.76)	0.13	1.51 (1.11–2.06)	0.009
Model 4^d^	1.20 (0.86–1.68)	0.28	1.29 (0.93–1.78)	0.13	1.53 (1.12–2.09)	0.007

*Note:* The second quartile (Q2) was selected as the reference (*n =* 405). Hypersensitive C-reactive protein was log-transformed

*Abbreviations: Q1 to Q4* lowest to highest quartile, *HR* hazard ratio, *CI* confidence interval, *CV* cardiovascular

^a^ Adjusted for age and sex

^b^ Adjusted for model 1 covariates and smoking, a history of cardiovascular events, diabetes, body mass index, and systolic blood pressure

^c^ Adjusted for model 2 covariates and hemoglobin, blood platelet count, serum potassium, serum albumin, low-density lipoprotein cholesterol, high-density lipoprotein cholesterol, hypersensitive C-reactive protein, and estimated glomerular filtration rate levels

^d^ Adjusted for model 3 covariates and the use of antiplatelet agents and lipid-lowering drugs

**Fig. 3:**
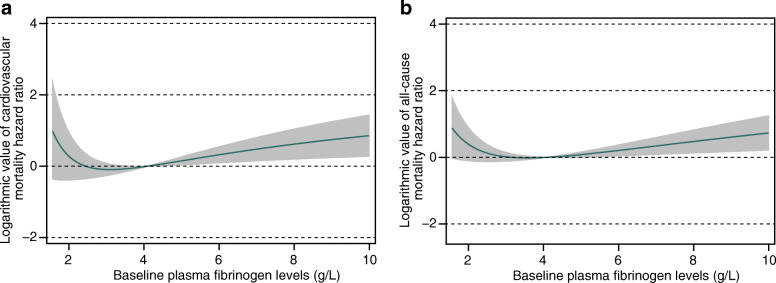
Nonlinear relationship between fibrinogen and cardiovascular (**a**) and all-cause (**b**) mortality. Multivariate adjusted logarithmic value of hazard ratios of cardiovascular and all-cause mortality associated with plasma fibrinogen levels in a Cox regression model using restricted cubic splines, adjusted for age, sex, smoking, a history of cardiovascular events, diabetes, body mass index, systolic blood pressure, hemoglobin, blood platelet count, serum potassium, serum albumin, low-density lipoprotein cholesterol, high-density lipoprotein cholesterol, hypersensitive C-reactive protein, estimated glomerular filtration rate, and the use of antiplatelet agents and lipid-lowering drugs. Hypersensitive C-reactive protein was log-transformed

Additionally, when we examined plasma fibrinogen as a continuous variable in multivariable adjusted cubic spline models, we found that the relationship between fibrinogen and CV and all-cause mortality was nonlinear, exhibiting approximate J-shaped curves (Fig. 3).

## Discussion

In this study, an elevated plasma fibrinogen level was significantly associated with an increased risk of CV and all-cause mortality in patients undergoing PD, and the relationship between plasma fibrinogen and mortality was nonlinear.

We found that the average plasma fibrinogen level in PD patients was higher than the normal range. There are some possible explanations for this phenomenon. First, with the loss of albumin in the peritoneal dialysate, free fatty acids accumulate in the blood, and these are strong stimulating factors for fibrinogen synthesis by the liver [24, 25]. Second, long-term and continuous exposure to glucose-based dialysate can produce significant metabolic disorders such as hyperinsulinemia, metabolic syndrome, and dyslipidemia, leading to endothelial dysfunction, inflammation, and prothrombotic tendency. Thus, PD patients showed an elevated fibrinogen level and a more prothrombotic profile than HD patients [20, 21]. In addition, with the decline of renal function, the clearance of fibrinogen and other procoagulant proteins by the kidneys is weakened [11, 12].

Our study found that high plasma fibrinogen levels were significantly associated with CV and all-cause mortality, which is consistent with some studies conducted on stage 3–4 CKD and HD patients [14, 16]. Studies have shown that in CKD patients, the expression level of tissue factor, the key initiating factor of the coagulation cascade, is increased, and the exogenous coagulation pathway is activated [26, 27]. When thrombin is formed, fibrinogen is converted into fibrin, which is mediated by factor XIII to form a cross-linked network around the platelet plug. The size of the fibrin fibers and the density of the overall clot are directly related to the level of fibrinogen [20]. Due to the long-term and continuous exposure to glucose-based dialysate, PD patients are prone to develop advanced glycation end products coupled with chronic inflammation and oxidized stress [11–13, 20]. In this case, fibrinogen becomes glycated and later oxidized through post-translational modifications [17, 28]. Some studies have investigated the effect of nonenzymatic glycosylation and the oxidation of fibrinogen on the properties of clots. Nonenzymatic glycosylation and oxidized fibrinogen may reduce clot permeability [29], increase fiber density, or decrease porosity coupled with a decreased individual fiber diameter, leading to an increase in the proportion of thin fibers and the formation of stiffer clots, which are less sensitive to plasmin and more difficult to lyse [30–33]. An increased clot density due to elevated fibrinogen levels has been reported to be independently and strongly associated with CV and all-cause mortality in dialysis patients [17]. This explanation is supported by the fact that in diabetic patients, fibrinogen has been shown to be glycated to form a denser clot and become resistant to fibrinolysis [34–36], and with the improvement of blood glucose control, the glycation of fibrinogen is reduced and the properties of clots are improved, including a decrease in lateral aggregation, an increase in permeability and the rate of fibrinolysis, and a decrease in the proportion of thin fibers in the overall clot [37]. In addition, post-translational modifications of fibrinogen include guanidinylation as well as glycation, which can also change the properties of clots. In particular, it can reduce the diameter of fibers, increase the formation of thin fibers and stiffer clots, and prolong the fibrinolysis time, thereby increasing the risk of thromboembolism morbidity and mortality [17].

Shlipak et al. [15] found that fibrinogen failed to predict adverse outcomes in individuals with CKD. Their conclusions contradict ours. This may be because, first, their cohort was different, and they investigated patients with a broader spectrum of CKD stages, while we focused only on PD patients. Second, in their study, the upper quartile was compared with the other lower 3 quartiles; therefore, it is possible to underestimate or obscure the complex U-shaped or J-shaped relationship between fibrinogen and mortality. Low fibrinogen levels can lead to an increased risk of bleeding, which also increases the risk of CV event morbidity and mortality [38]. Moreover, the relationships between some nontraditional CV risk factors, such as albumin and hemoglobin, and mortality are nonlinear [14]. Therefore, we further examined the relationship between plasma fibrinogen and mortality by using an adjusted restricted cubic spline method, and we discovered that the relationship between fibrinogen and CV and all-cause mortality was nonlinear, exhibiting approximate J-shaped curves. However, we did not find that lower fibrinogen levels were associated with CV and all-cause mortality. This is probably because bleeding is multifactorial and can be attributed to thrombocytopenia, uremic disturbance of platelet adhesion and aggregation, morphological changes of the vessels, anemia, coagulation, and fibrinolysis [39–41]. Interactions among multiple factors may mask the effect of the fibrinogen level alone on the prognosis of PD patients.

Our study has several limitations. First, we studied data only at the baseline level and did not consider the impact of data changes on outcomes during the follow-up period. Second, we did not include platelet function, clot lysis time, Kt/V, or other factors that may be associated with thrombosis and prognosis, and it is difficult to rule out all residual confounding factors. Third, relying on expert consensus and death certificates to determine the cause of death may lead to misclassification. Finally, all participants came from the Chinese population, limiting its generalization to other ethnic groups.

## Conclusions

In summary, an elevated plasma fibrinogen level was significantly associated with an increased risk of CV and all-cause mortality in patients undergoing PD, and the relationship between plasma fibrinogen and mortality was nonlinear. These findings provide new insights into the effects of fibrinogen on the prognosis of PD patients. In the future, we need to evaluate whether reducing fibrinogen levels can improve the prognosis of PD patients and clarify the underlying mechanisms.

## Supplementary information

**Additional file 1: Table S1.** Significant influencing factors for plasma fibrinogen levels in a simple linear regression.

## Data Availability

The datasets used and/or analyzed during the current study are available from the corresponding author on reasonable request.

## Abbreviations

CV: Cardiovascular
PD: Peritoneal dialysis
HR: Hazard ratio
CI: Confidence interval
ESRD: End-stage renal disease
hs-CRP: Hypersensitive C-reactive protein
HD: Hemodialysis
CKD: Chronic kidney disease
BMI: Body mass index
TC: Total cholesterol
TG: Triglycerides
LDL-C: Low-density lipoprotein cholesterol
HDL-C: High-density lipoprotein cholesterol
eGFR: Estimated glomerular filtration rate
CKD-EPI: Chronic Kidney Disease Epidemiology Collaboration
ANOVA: One-way analysis of variance
SBP: Systolic blood pressure
DBP: Diastolic blood pressure
