# Supplementary material for: Plasma fibrinogen and mortality in patients undergoing peritoneal dialysis: a prospective cohort study
Source: BMC Nephrol. 2020 Aug 17;21:349. doi: 10.1186/s12882-020-01984-6 (PMC7430005; doi:10.1186/s12882-020-01984-6)
Supplement: Supplementary file 1 — Additional file 1: Table S1. Significant influencing factors for plasma fibrinogen levels in a simple linear regression. [file 12882_2020_1984_MOESM1_ESM.docx]

**Additional file 1.**

**Table S1** Associated factors of plasma fibrinogen levels in a simple linear regression

| Variables | Unstandardized  coefficients | | Standardized coefficients | t | *P* |
| --- | --- | --- | --- | --- | --- |
|  | B | Standard error | β |  |  |
| Age (years) | 0.02 | 0.002 | 0.20 | 7.96 | < 0.001 |
| Sex (female/male) | −0.20 | 0.07 | −0.07 | −2.81 | 0.005 |
| Smoker (yes/no) | 0.28 | 0.09 | 0.08 | 3.28 | 0.001 |
| History of CV events (yes/no) | 0.56 | 0.09 | 0.16 | 6.54 | < 0.001 |
| Diabetes (yes/no) | 0.79 | 0.08 | 0.25 | 10.29 | < 0.001 |
| Hypertension (yes/no) | −0.13 | 0.11 | −0.03 | −1.23 | 0.22 |
| BMI (kg/m^2^) | 0.10 | 0.01 | 0.24 | 9.55 | < 0.001 |
| SBP (mmHg) | 0.004 | 0.001 | 0.07 | 2.60 | 0.009 |
| DBP (mmHg) | −0.004 | 0.002 | −0.04 | −1.76 | 0.08 |
| Hemoglobin (g/L) | 0.002 | 0.002 | 0.03 | 1.16 | 0.25 |
| Blood platelet count (per 10×10^9^/L greater) | 0.06 | 0.004 | 0.33 | 13.76 | < 0.001 |
| Serum potassium (mmol/L) | −0.14 | 0.04 | −0.09 | −3.49 | < 0.001 |
| Serum albumin (g/L) | −0.05 | 0.007 | −0.18 | −7.45 | < 0.001 |
| TC (mmol/L) | 0.15 | 0.02 | 0.15 | 6.21 | < 0.001 |
| TG (mmol/L) | 0.19 | 0.03 | 0.14 | 5.71 | < 0.001 |
| LDL-C (mmol/L) | 0.22 | 0.03 | 0.17 | 6.86 | < 0.001 |
| HDL-C (mmol/L) | −0.43 | 0.11 | −0.10 | −4.12 | < 0.001 |
| hs-CRP (per log-unit greater) | 0.29 | 0.02 | 0.31 | 11.97 | < 0.001 |
| eGFR (mL/min/1.73 m^2^) | 0.002 | 0.01 | 0.004 | 0.17 | 0.87 |
| Use of antiplatelet agents (yes/no) | 0.55 | 0.11 | 0.13 | 5.05 | < 0.001 |
| Use of lipid-lowering drugs (yes/no) | 0.56 | 0.11 | 0.13 | 5.20 | < 0.001 |

*Note:* Hypersensitive C-reactive protein was log-transformed

*Abbreviations*: *CV* cardiovascular, *BMI* body mass index, *SBP* systolic blood pressure, *DBP* diastolic blood pressure, *TC* total cholesterol, *TG* triglycerides, *LDL-C* low-density lipoprotein cholesterol, *HDL-C* high-density lipoprotein cholesterol, *hs-CRP* hypersensitive C-reactive protein, *eGFR* estimated glomerular filtration rate
